# The MELD-Plus: A generalizable prediction risk score in cirrhosis

**DOI:** 10.1371/journal.pone.0186301

**Published:** 2017-10-25

**Authors:** Uri Kartoun, Kathleen E. Corey, Tracey G. Simon, Hui Zheng, Rahul Aggarwal, Kenney Ng, Stanley Y. Shaw

**Affiliations:** 1 Harvard Medical School, Boston, Massachusetts, United States of America; 2 Center for Systems Biology; Center for Assessment Technology & Continuous Health (CATCH), Massachusetts General Hospital, Boston, Massachusetts, United States of America; 3 Gastrointestinal Unit, Massachusetts General Hospital, Boston, Massachusetts, United States of America; 4 Center for Biostatistics, Massachusetts General Hospital, Boston, Massachusetts, United States of America; 5 IBM Research, Cambridge, Massachusetts, United States of America; Chang Gung Memorial Hospital Kaohsiung Branch, TAIWAN

## Abstract

**Background and aims:**

Accurate assessment of the risk of mortality following a cirrhosis-related admission can enable health-care providers to identify high-risk patients and modify treatment plans to decrease the risk of mortality.

**Methods:**

We developed a post-discharge mortality prediction model for patients with a cirrhosis-related admission using a population of 314,292 patients who received care either at Massachusetts General Hospital (MGH) or Brigham and Women’s Hospital (BWH) between 1992 and 2010. We extracted 68 variables from the electronic medical records (EMRs), including demographics, laboratory values, diagnosis codes, and medications. We then used a regularized logistic regression to select the most informative variables and created a risk score that comprises the selected variables. To evaluate the potential for generalizability of our score, we applied it on all cirrhosis-related admissions between 2010 and 2015 at an independent EMR data source of more than 18 million patients, pooled from different health-care systems with EMRs. We calculated the areas under the receiver operating characteristic curves (AUROCs) to assess prediction performance.

**Results:**

We identified 4,781 cirrhosis-related admissions at MGH/BWH hospitals, of which 778 resulted in death within 90 days of discharge. Nine variables were the most effective predictors for 90-day mortality, and these included all MELD-Na’s components, as well as albumin, total cholesterol, white blood cell count, age, and length of stay. Applying our nine-variable risk score (denoted as “MELD-Plus”) resulted in an improvement over MELD and MELD-Na scores in several prediction models. On the MGH/BWH 90-day model, MELD-Plus improved the performance of MELD-Na by 11.4% (0.78 [95% CI, 0.75–0.81] versus 0.70 [95% CI, 0.66–0.73]). In the MGH/BWH approximate 1-year model, MELD-Plus improved the performance of MELD-Na by 8.3% (0.78 [95% CI, 0.76–0.79] versus 0.72 [95% CI, 0.71–0.73]). Performance improvement was similar when the novel MELD-Plus risk score was applied to an independent database; when considering 24,042 cirrhosis-related admissions, MELD-Plus improved the performance of MELD-Na by 16.9% (0.69 [95% CI, 0.69–0.70] versus 0.59 [95% CI, 0.58–0.60]).

**Conclusions:**

We developed a new risk score, MELD-Plus that accurately stratifies the short-term mortality of patients with established cirrhosis, following a hospital admission. Our findings demonstrate that using a small set of easily accessible structured variables can help identify novel predictors of outcomes in cirrhosis patients and improve the performance of widely used traditional risk scores.

## Introduction

Cirrhosis-related complications account for 1.1% and 1.8% of all deaths in the United States and Europe, respectively [1, 2]. In addition to increased mortality, individuals with cirrhosis suffer from significantly worse health issues and greater disability compared to those without cirrhosis [3].

Although risk-stratification tools for the prediction of cirrhosis-related mortality are available [4–9], these models are based on small populations and use a limited number of preselected traditional predictors. Improved mortality prediction scores may highlight the clinical variables that contribute to mortality risk, including modifiable factors, and guide the allocation of resources to improve cirrhosis care for high-risk patients.

The recent availability of large cohorts of data from electronic medical records (EMRs) allows for the development of improved mortality prediction scores through inclusion of a broader set of clinically applicable, unbiased variables. Not only that, but developing such prediction models allows clinicians to identify the clinical variables that contribute to mortality risk, including modifiable factors. Improving current standard models like the model for end-stage liver disease (MELD) and MELD-Na can guide clinicians in better targeting treatment to improve cirrhosis care and outcomes for high-risk patients.

Cohorts assembled from EMRs represent a powerful resource to study disease complications at the population level. Recent studies have demonstrated the usefulness of EMR analysis to discover or confirm outcome correlations, sub-categories of disease, and adverse drug events [10–14]. The MELD score is based on three commonly used laboratory tests available in the EMRs, and it is the most widely used tool to predict outcomes in patients with cirrhosis [15, 16]. An extended version of MELD, one that incorporates serum sodium levels, the MELD-Na score, has been recently adopted by The Liver and Intestine Transplantation Committee for liver transplant allocation [17]. Although the two scores are simple to calculate and apply in a practical sense, the improved accessibility of a wide variety of variables from EMRs raises the possibility that prediction models could benefit from the inclusion of a broader, unbiased set of clinical variables. Identifying a combination of the most informative variables may improve the prognostic utility beyond that of current risk scores.

The aim of the present study was to develop a risk score to predict mortality following a cirrhosis-related admission. We demonstrated that a score composed of a small set of easily accessible clinical variables improves the prediction performance of both the MELD and MELD-Na scores. We further demonstrated the generalizability of our model through independent validation in a large EMR-based data source.

## Methods

### Study population

We analyzed a previously defined cohort of 314,292 patients at increased risk for metabolic disease who were admitted to Massachusetts General Hospital (MGH) or Brigham and Women’s Hospital (BWH*)* between 1992 and 2010 [13]. We identified an admission as cirrhosis*-*related when the keyword “cirrhosis” was present in the discharge summary of the admission and we observed at least one ICD-9 code (571.2, 571.5, or 571.6 as in [18]) within the 30 days preceding the discharge date, including during the admission. This identification method was validated by a physician (Dr. Kathleen Corey) chart review.

We excluded elective admissions if they included at least one diagnosis or procedure code for liver biopsy, radiofrequency ablation, transarterial chemoembolization, hepatic resection, or liver transplant. We included only patients 18 years of age or older at the time of the admission, and we tracked the records of all patients for 90 days after their discharge. We determined mortality through linkage to the social security master death index.

### Prediction modeling

To predict mortality within 90 days, we developed a model that included a large set of structured variables extracted from the EMRs. In addition to variables available during the period of admission, we considered variables available for the period of 12 months preceding the discharge date (see Table 1).

**Table 1:** Baseline characteristics. All values extracted during the 12 months preceding discharge date. For laboratory variables, values are the most recent. Comorbidity calculations count the number of diagnosis codes. Prevalence calculations consider admissions with at least one measurement for laboratories and at least one diagnosis code for comorbidities.

Variable and category	Cirrhosis-related admissions (n = 4,781)
**Age (years); Mean (SD)**	60.0 (13.7)
**Gender (%)**	
Male	64.4
Female	35.6
**Ethnicity (%)**	
Caucasian	77.4
African American	6.8
Other	2.3
Unknown	13.5
**Insurance Type (could be ≥ 1 types per patient) (%)**	
Medicaid	5.6
Medicare	60.0
Other	98.6
**BMI (kg/m**^**2**^**); Mean (SD)**	28.7 (8.2)
**Laboratory values; Mean (SD) / Prevalence (%)**	
Sodium (mmol/l)	136.5 (5.8) / 99.7
eGFR (ml/min/1.73m^2^)	59.6 (33.7) / 31.9
WBC (th/cumm)	6.7 (3.8) / 99.8
Platelets (th/cumm)	141.3 (100.0) / 99.8
Prothrombin time (INR)	1.5 (0.5) / 91.3
Albumin (g/dl)	2.9 (0.7) / 98.7
Total Bilirubin (mg/dl)	2.5 (4.3) / 98.7
Transaminase SGOT (u/l)	60.2 (76.6) / 98.8
Transaminase SGPT (u/l)	36.1 (38.2) / 96.7
GGT (u/l)	249.4 (346.6) / 6.8
MELD score	14.21 (6.1) / 84.8
NAFLD Fibrosis score	1.70 (2.1) / 13.7
**Comorbidities; Mean (SD) / Prevalence (%)**	
Variceal hemorrhage / Gastrointestinal bleed	0.8 (2.1) / 24.2
Spontaneous bacterial peritonitis	0.1 (0.6) / 3.4
Hepatocellular carcinoma	0.7 (5.5) / 4.3
Hepatorenal syndrome	0.1 (0.5) / 3.2
Hepatic encephalopathy	1.4 (3.6) / 28.6
Ascites	2.4 (5.7) / 37.9
Renal failure	1.8 (8.4) / 13.1
Cerebrovascular disease	0.5 (2.4) / 10.8
Congestive heart failure	2.7 (7.9) / 36.7
Hypertension	3.1 (7.2) / 58.9
Acute myocardial infarction	0.5 (2.1) / 13.4
Ischemic heart disease	0.3 (1.2) / 12.4
Peripheral vascular disease	0.5 (2.2) / 13.0
Diabetes	5.9 (10.3) / 57.0

The variables included demographics (e.g., gender, ethnicity, marital status), laboratory measurements (e.g., albumin, sodium), and medications (e.g., anticoagulants, lipid lowering agents). For laboratory variables, we used the most recent values found during admission (when no value was found during admission, we considered the preceding 12 months). Typically, common laboratory measurements were available during the admission (as seen in Table 1). We determined comorbidities from the number of diagnosis codes within the 12 months prior to the discharge date, and we determined medication count by recording the number of prescriptions within the 12 months preceding the discharge date.

Additional variables included body mass index, NAFLD fibrosis score (Eq 1), and the MELD score (Eq 2). Missing values were imputed with the mean of the available data for each variable. We randomly selected two thirds of the admissions to serve as a derivation set, whereas the remaining one third served as a validation set. A complete list of the variables we used is available in S1 Table, and all diagnoses and procedure definitions used in this study are available in S2 Table.

$$\begin{array}{l} \mathrm{NAFLD}\;\mathrm{Fibrosis}\;\mathrm{Score}=\text{-}\;1.675+0.037\times \mathrm{Age}+0.094\times \mathrm{BMI}\\ +1.13\times \mathrm{IFG}/\mathrm{Diabetes}\;(\mathrm{yes}=1,\mathrm{no}=0)\\ +0.99\;\times \mathrm{AST}/\mathrm{ALT}\;\mathrm{ratio}\;\text{-}\;0.013\;\times \mathrm{Platelet}\;\text{-}\;0.66\times \mathrm{Albumin} \end{array}$$ (1)

$$\begin{array}{l} \mathrm{MELD}\;\mathrm{Score}=+6.43+9.57\times \ln(\mathrm{Creatinine})\\ +3.78\times \ln(\mathrm{Total}\;\mathrm{Bilirubin})+11.2\times \ln(\mathrm{INR}) \end{array}$$ (2)

To select the most informative variables, we applied feature selection on the derivation set. We used logistic regression with the adaptive least absolute shrinkage and selection operator (LASSO) algorithm [19] because it is considered an efficient algorithm for parsimoniously ranking variables in clinical predictive modeling [20, 21]. We considered all variables that were statistically significantly different from a univariate analysis (*P* < 0.05) as in [22]. The generalized linear model (GLM) equations used to calculate prediction risk at MGH/BWH and at the independent EMR source are presented in Eqs 3 and 4.

$$\begin{array}{l} L=\\ +11.794383\;\\ +2.076192\times \mathrm{Log}10(1+\mathrm{Total}\;\mathrm{Bilirubin})\\ +2.494291\times \mathrm{Log}10(1+\mathrm{Creatinine})\\ \text{-}\;6.049540\times \mathrm{Log}10(1+\mathrm{Albumin})\\ +2.525904\times \mathrm{Log}10(1+\mathrm{INR})\\ +1.911856\times \mathrm{Log}10(1+\mathrm{WBC})\\ +0.015411\times \mathrm{Length}\;\mathrm{of}\;\mathrm{stay}\\ \left(\mathrm{number}\;\mathrm{of}\;\mathrm{nights}\;\mathrm{the}\;\mathrm{patient}\;\mathrm{spent}\;\mathrm{in}\;\mathrm{the}\;\mathrm{hospital}\;\mathrm{during}\;\mathrm{the}\;\mathrm{cirrhosis}\text{-}\mathrm{related}\;\mathrm{admission}\right)\\ +0.041047\times \mathrm{Age}\;(\mathrm{years})\\ \text{-}\;6.625270\times \mathrm{Log}10(1+\mathrm{Sodium})\\ \text{-}\;1.445666\times \mathrm{Log}10(1+\mathrm{Total}\;\mathrm{Cholesterol}) \end{array}$$ (3)

$$\mathrm{MELD}\text{-}\mathrm{Plus}=p(90\;\mathrm{day}\;\mathrm{mortality})=\frac{\exp(L)}{1+\exp(L)}$$ (4)

To calculate 95% confidence intervals, we applied the bootstrap procedure with 1,000 replicates. We calculated the area under the receiver operating characteristic curves (AUROC) to measure the model’s accuracy in the validation set. Additionally, we evaluated for overfitting by comparing the AUROC in the validation set to an average AUROC value for 100 permutations of randomly selected derivation and validation sets (each including two thirds and one third of the derivation set’s cirrhosis-related admissions, respectively). We compared categorical variables using a chi-squared test, and we compared the differences in the means of continuous variables using a t-test or Wilcoxon rank sum test, as appropriate. We further compared the differences in standard deviations by using an F-test. All statistical tests were two-sided, with Bonferroni corrections for the 68 comparisons, and the adjusted *P* value was 7.4∙10^−4^ for each comparison. We performed all programming using the R statistical language [23].

### Independent validation

We were granted access to a data source of 18,345,793 individuals, pooled from multiple different health-care systems with EMRs (“The IBM Explorys Network”) [24]. The data were standardized and normalized using common ontologies, searchable through a HIPAA-enabled, de-identified cloud-computing platform. Patients were seen in multiple health-care systems between 2010 and 2015, with a combination of data from clinical EMRs, outgoing health-care system bills, and adjudicated payor claims.

We first identified all cirrhosis-related admissions in this database, and then we extracted values for the selected variables of our MGH/BWH 90-day model. We further deployed the GLM equations on the independent source (Eqs 3 and 4). Missing values were imputed based on the mean values of the MGH/BWH 4,781 cirrhosis-related admissions: total bilirubin (2.486604493), creatinine (1.375274633), albumin (2.888940902), INR (1.499619615), WBC (6.673836966), sodium (136.5401552), and total cholesterol (133.6246914).

Because death dates were not available for patients in the IBM Explorys Network, we used the year of death to determine the outcome. We were able then only to use a 1-year estimated death for this population (e.g., for a patient discharged on October 13, 2010, we could only determine if the patient died either in 2010 or 2011, or survived after that). To compare the performance of the IBM Explorys approximate one-year prediction model, we used the original 314,292-patient population at MGH/BWH and applied the same approximate one-year mortality outcome identification method. We calculated AUROCs for MELD, MELD-Na (Eq 5), and for our risk score (Eqs 3 and 4).

MELD-Na=MELD+1.59×(135-Na) (5)

The institutional review board of Partners HealthCare and IBM approved this study and all its methods, including the EMR cohort assembly, data extraction, and analyses.

## Results

### Univariate analysis

We identified a total of 4,781 admissions as cirrhosis-related, of which 778 resulted in death within 90 days of the discharge date (16.3%). In a sample of 50 randomly selected patients, 64% were admitted primarily for cirrhosis, for instance, due to the presence of ascites or spontaneous bacterial peritonitis, and the rest had the comorbidity of cirrhosis but were admitted primarily for different reasons such as heart failure or chronic obstructive pulmonary disease (COPD). Individuals who died within the 90-day period after discharge were older in comparison with those who survived (64.1 years versus 59.2 years, *P* = 4.22∙10^−17^); however, the two populations did not differ by gender (65.0% male versus 64.0% female, *P* = 1.0) or ethnicity (77.0% Caucasian for both, *P* = 1.0).

We calculated event ratios by dividing the values ascertained for the two populations (e.g., the mean MELD scores were 18.5 and 13.3 for admissions that resulted in death and survival, respectively, yielding a ratio of 1.4, *P* = 4.45∙10^−65^). Individuals who died within the 90-day period after discharge had higher ratios of liver-related comorbidities than those who survived, and these comorbidities included hepatorenal syndrome (ratio = 5.1, *P* = 4.60∙10^−21^), hepatocellular carcinoma (ratio = 5.0, *P* = 1.40∙10^−16^), and ascites (ratio = 2.1, *P* = 3.55∙10^−24^). Laboratory measurements also significantly differentiated the two populations. For instance, albumin was lower in those who died within the 90-day period (2.60 g/dl versus 2.95 g/dl, *P* = 5.64∙10^−35^), and the total bilirubin (4.87 mg/dl versus 2.02 mg/dl, *P* = 1.82∙10^−41^), INR (1.70 versus 1.46, *P* = 5.63∙10^−30^), and creatinine (1.80 mg/dl versus 1.29 mg/dl, *P* = 1.24∙10^−36^) were higher in those who died within the 90-day period.

No difference was found in the prevalence of COPD, cerebrovascular disease, diabetes, coronary artery disease, peripheral vascular disease, pneumonia, or sleep apnea between the populations. The complete list of variables, indicating the differences between the surviving and the deceased populations, is presented in S3 Table.

### Logistic regression model

**Fig 1:**
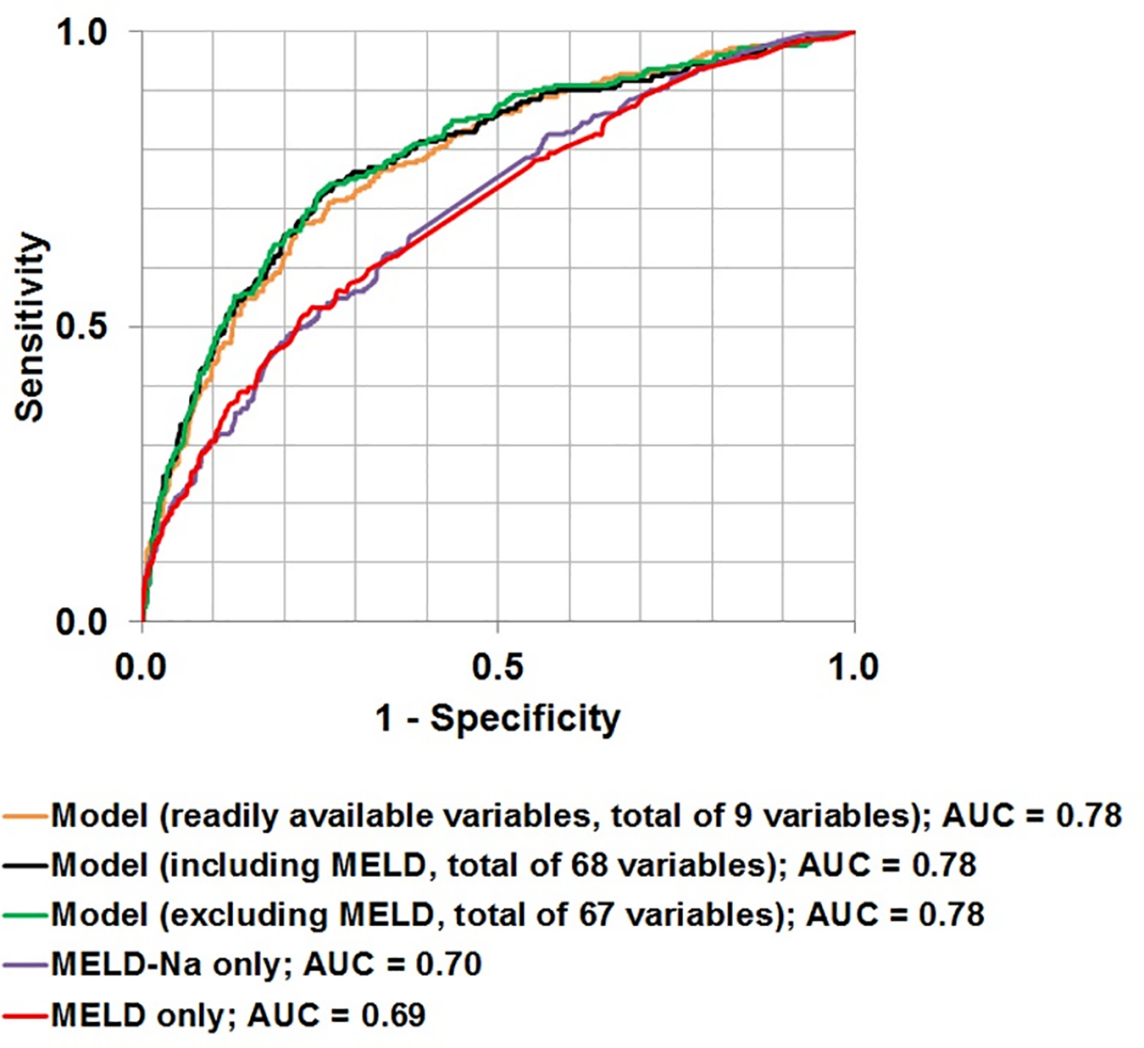
AUROCs using differing variable combinations in a 90-day mortality prediction model at MGH/BWH.

The AUROCs of 0.78 were identical for all three models composed of multiple variables (Fig 1). With generalizability in mind and the potential ease of extraction of commonly available laboratory values and other trivial variables (e.g., age, length of stay), we decided to follow the model that comprised the 9 readily available clinical variables. To evaluate the contribution of the MELD score to the 90-day mortality prediction, we evaluated the performance of MELD and MELD-Na scores alone. Considering the 4,781 admissions, using the MELD score alone to predict the 90-day mortality resulted in an AUROC value of 0.69. An additional model using the MELD-Na score alone yielded an AUROC value of 0.70.

Each of the MELD-Na components were associated with an increased mortality, including INR (OR, 1.58; 95% CI, 1.30–1.96), creatinine (OR, 1.25; 95% CI, 1.16–1.34), total bilirubin (OR, 1.11; 95% CI, 1.08–1.14), and sodium (OR, 0.97; 95% CI, 0.95–0.99). Other laboratory measurements associated with mortality included WBC (OR, 1.10; 95% CI, 1.07–1.13), total cholesterol (OR, 0.996; 95% CI, 0.993–0.999), and albumin (OR, 0.45; 95% CI, 0.37–0.52). Additional predictors included age at time of the admission (OR, 1.04; 95% CI, 1.03–1.05) and length of stay (OR, 1.02; 95% CI, 1.005–1.03).

Because total cholesterol and hospital length of stay are typically not uniform factors across different hospitals and may vary in different countries, we evaluated an additional model that included only 7 of the 9 variables. This yielded an AUROC of 0.77 and resulted in the following associations with increased mortality: INR (OR, 1.66; 95% CI, 1.38–2.05), creatinine (OR, 1.25; 95% CI, 1.17–1.35), total bilirubin (OR, 1.11; 95% CI, 1.08–1.14), sodium (OR, 0.97; 95% CI, 0.95–0.98), WBC (OR, 1.10; 95% CI, 1.07–1.13), albumin (OR, 0.43; 95% CI, 0.36–0.51), and age (OR, 1.04; 95% CI, 1.03–1.05). We present the GLM equations used to calculate prediction performance at MGH/BWH in Eqs 6 and 7.

$$\begin{array}{l} L=\\ +8.53499496\;\\ +2.06503238\times \mathrm{Log}10(1+\mathrm{Total}\;\mathrm{Bilirubin})\\ +2.59679650\times \mathrm{Log}10(1+\mathrm{Creatinine})\\ \text{-}\;6.34990436\times \mathrm{Log}10(1+\mathrm{Albumin})\\ +2.99724802\times \mathrm{Log}10(1+\mathrm{INR})\\ +1.92811726\times \mathrm{Log}10(1+\mathrm{WBC})\\ +0.04070442\times \mathrm{Age}\;(\mathrm{years})\\ \text{-}\;6.47834101\times \mathrm{Log}10(1+\mathrm{Sodium}) \end{array}$$ (6)

$$\begin{array}{l} \mathrm{MELD}\text{-}\mathrm{Plus}\;(\mathrm{excluding}\;\mathrm{length}\;\mathrm{of}\;\mathrm{stay}\;\mathrm{and}\;\mathrm{total}\;\mathrm{cholesterol})=\\ p(90\;\mathrm{day}\;\mathrm{mortality})=\frac{\exp(L)}{1+\exp(L)} \end{array}$$ (7)

### Prediction of 90-day mortality after a cirrhosis-related admission

**Fig 2:**
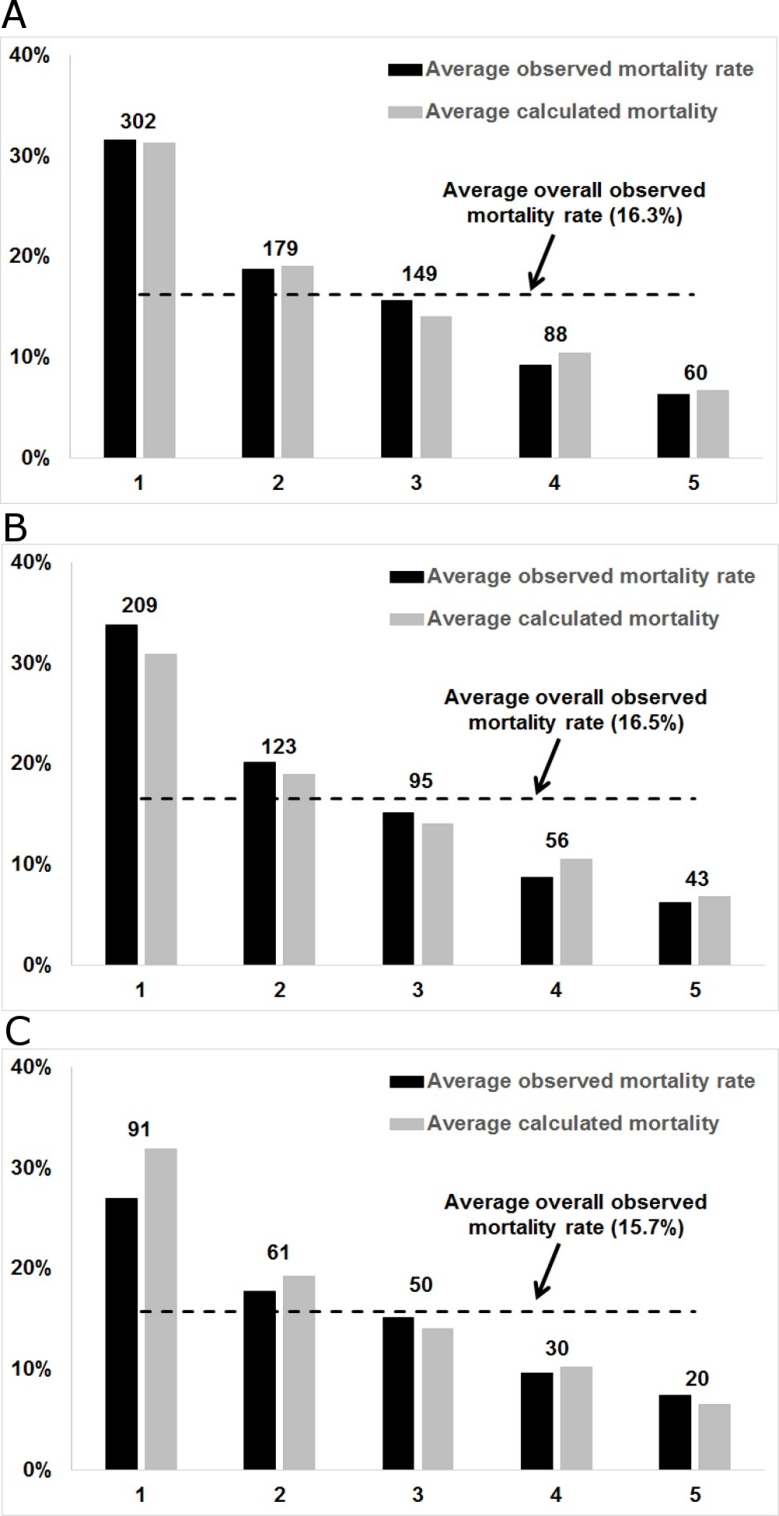
Predicted versus observed 90-day mortality within each risk quintile. (A) Entire cohort of 4,781 cirrhosis-related admissions. (B) Derivation set of 3,187 cirrhosis-related admissions. (C) Validation set of 1,594 cirrhosis-related admissions.

Using our 9-variable risk score, we divided our population into quintiles and compared the average predicted 90-day mortality with the observed mortality within each quintile. The predicted 90-day mortality derived from a logistic regression model for each admission and indicated the probability that a patient who survived the admission would die within 90 days post discharge. As shown in Fig 2A–2C, the predicted 90-day mortality was strongly correlated with the observed mortality rate throughout the range of risk in both derivation and validation sets (Kendall’s *τ* = 1.0; *P* = 0.027; Pearson correlation *r* = 0.995 for the correlation between the average calculated and observed mortality). We provide the logistic regression equations used to calculate the predicted 90-day mortality probabilities in Eqs 3 and 4. The complete list of variables that indicate the differences between the highest-risk quantile and the lowest-risk quantile populations are presented in S4 Table.

### Generalization evaluation

**Fig 3:**
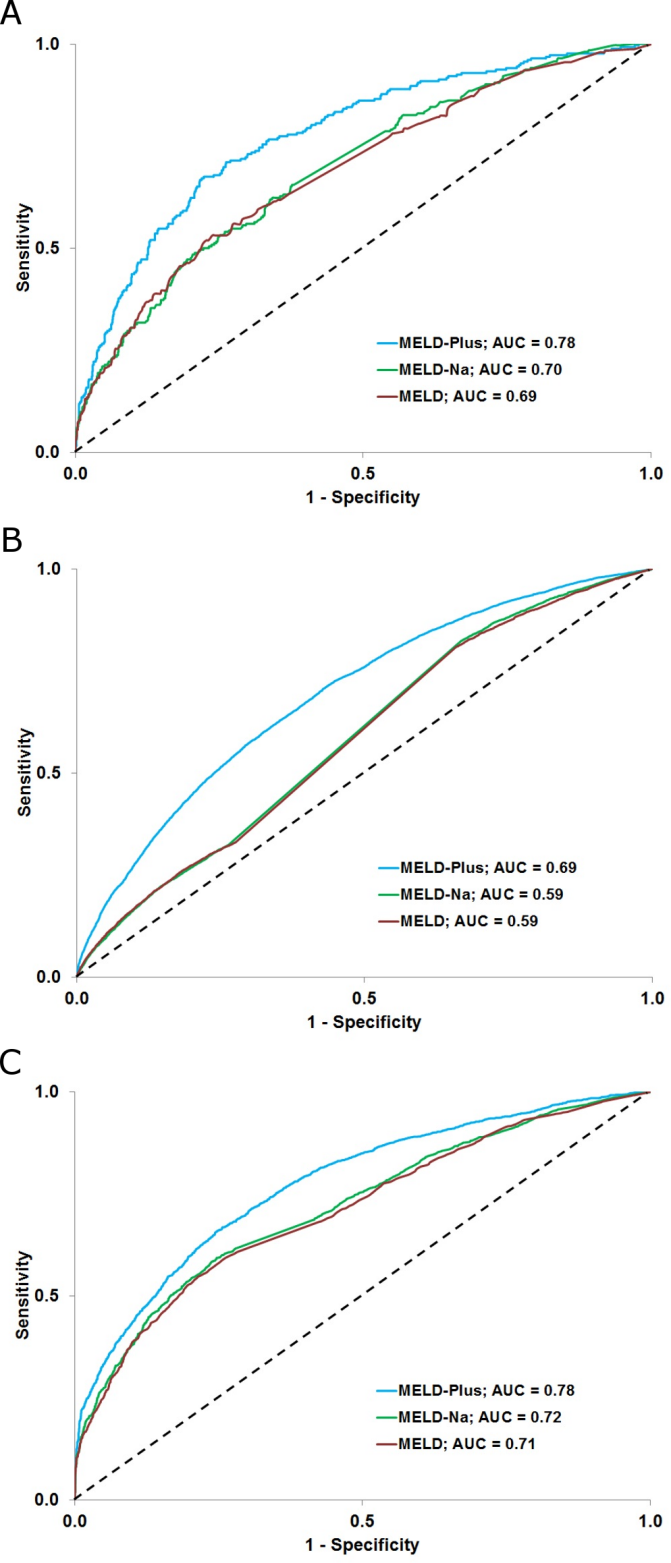
Prediction performance across different cirrhosis populations. (A) MGH/BWH 90-day mortality (4,781 cirrhosis-related admissions). (B) The IBM Explorys Network approximate 1-year mortality (24,042 cirrhosis-related admissions). (C) MGH/BWH approximate 1-year mortality (4,680 cirrhosis-related admissions).

Applying our 9-variable risk score (the MELD-Plus score) demonstrated an improvement over MELD and MELD-Na scores in all prediction models, as shown in Fig 3. On the MGH/BWH 90-day model, MELD-Plus improved the performance of MELD-Na by 11.4% (0.78 [95% CI, 0.75–0.81] versus 0.70 [95% CI, 0.66–0.73]). On the MGH/BWH approximate 1-year model, MELD-Plus improved the performance of MELD-Na by 8.3% (0.78 [95% CI, 0.76–0.79] versus 0.72 [95% CI, 0.71–0.73]). On the IBM Explorys Network model used for external validation, MELD-Plus improved the performance of MELD-Na by 16.9% (0.69 [95% CI, 0.69–0.70] versus 0.59 [95% CI, 0.58–0.60]).

It is notable that the performance of MELD-Plus on the IBM Explorys data was lower in comparison with both MGH/BWH models (0.69 versus 0.78). Consistent with MELD-Plus, the performance of MELD and MELD-Na were also much lower on the IBM Explorys data in comparison with MGH/BWH. A potential reason for this is that the IBM Explorys Network population was relatively healthier. Patients in the IBM Explorys network had lower severity of liver disease in comparison with the corresponding MGH/BWH 1-year prediction model (mean MELD: 9.4 versus 16.8; *P* < 0.0001, mean MELD-Na: 11.4 versus 18.1; *P* < 0.0001). There may be other differences in the data or populations in the independent systems; the Partners HealthCare Research Patient Data Registry collected the MGH/BWH data, whereas dozens of distinct data aggregation mechanisms collected the data for the IBM Explorys Network. Furthermore, the variability in the levels of prediction performance might be influenced by the variability in the data; prediction performance might be higher when there is more variability in the data source (i.e., the population comprising patients with a broad spectrum of levels of cirrhosis severity). In the other direction, when the data is more uniform (e.g., most patients have just been diagnosed with cirrhosis for the first time, and only a minority suffers from an advanced cirrhosis), then prediction accuracy is lower. This hypothesis was confirmed because the IBM Explorys network had a statistically significant lower standard deviation of severity of liver disease in comparison with the MGH/BWH 1-year population (STD MELD: 1.8 versus 8.2; *P* < 0.0001, STD MELD-Na: 3.6 versus 8.2; *P* < 0.0001).

## Discussion

In this study, we used accessible EMR variables to develop a highly accurate, predictive model of 90-day post-discharge mortality in individuals with cirrhosis. We identified 9 variables that accurately predicted 90-day mortality with an AUROC of 0.78. Our risk score improved the performance of MELD and MELD-Na scores in multiple, independent patient populations, and this also held true in a large external validation patient cohort. Furthermore, our model’s calculated 90-day mortality risk was highly correlated with the observed mortality rate across all five risk quintiles. In particular, the model’s performance on the highest-risk quintile (the calculated and observed 90-day mortality was 31.6% and 31.2%, respectively) suggests that high-risk patients can be accurately identified. An additional model that included only 7 of the 9 variables and excluded length of stay and total cholesterol yielded an AUROC of 0.77 [95% CI, 0.74–0.80]. Although the 7-variable model demonstrated improved identification ability compared to MELD or MELD-Na, the improved prediction performance achieved by including total cholesterol in MELD-Plus suggests that it may be beneficial for cholesterol labs to be routinely collected in cirrhosis admission order sets.

The MELD score has been used extensively to predict patient outcomes, mortality, and readmission rates in individuals with cirrhosis [25, 26, 4]. Furthermore, although MELD-Na [17] was superior to MELD, the MELD-Plus score yielded improved levels of discrimination consistently in all prediction models, with AUROCs that significantly outperformed the traditional scores. These findings suggest that new types of cirrhosis-related risk indexes utilizing novel risk indicators may improve prognostication in this high-risk population.

MELD-Plus includes all MELD-Na’s components, as well as additional variables (albumin, total cholesterol, WBC, age, and length of stay). It is logical that a predication model that has all the MELD-Na model variables and additional ones would perform better, as was observed by MELD-Plus. Not only that, but many of the variables have physiological plausibility for inclusion in a prediction model. Decreased albumin correlated with worse outcomes in our model, which may be the result of decreased albumin marking decreased liver function in cirrhosis patients [25, 5]. Increasing age and length of hospital stay helped predict worse outcomes as well as could be expected. Along with that, higher WBC was correlated with a worse prognosis, potentially indicating poorer patient status (e.g., infection) at time of score calculation. Although patients may have multiple WBC measurements during admission, our model is both internally and externally valid because it uses the most recent WBC lab value. We chose the most recent WBC during model development because the last available set of labs is more reflective of the current health of patients than older measurements. Surprisingly, increased total cholesterol predicted a more favorable prognosis. Although unintuitive at first, this aligns with previous reports that claim cholesterol levels become less of a risk factor or even an inverse risk factor for mortality because serious diseases may lower cholesterol soon before death occurs [27].

Although our study describes analyses of retrospective medical databases, the proposed score could be used to identify patients that are at a high-risk of mortality in real time and thus may inform risk-stratification and therapeutic decision-making. In a desirable scenario, our score could be calculated automatically as an integrated component of an EMR system; the clinician would see a risk score (probability) or a risk quantile (highest, lowest, or in between) associated with the discharged patient, and this could be used to guide outpatient monitoring strategies. With further validation, the MELD-Plus score could also be used longitudinally in outpatients to monitor disease progression and/or responses to therapy.

Our study has limitations. First, it is a retrospective analysis limited to two academic, tertiary-care hospitals. Even though we validated our model on a large external patient cohort, subsequent studies must further assess the validity of our model in the external population and consider different age ranges, coding systems, and data-collection methods. Second, the cirrhosis populations may vary at different centers—for example, alcohol use might significantly vary between patients residing in the Boston area versus patients residing in other states [28]. Furthermore, although MGH and BWH are urban care facilities, the high prevalence of rural populations at the IBM Explorys Network might affect prediction performance. Third, although mortality was recorded, either through linkage to the social security master death index as in the MGH/BWH models or through using EMR or billing/claims in the IBM Explorys model, such death indications may under-represent the true mortality rates. To minimize this potential under-representation, we considered only patients who survived the study follow-up. All patients had EMR data entries (such as laboratory measurements) after the study follow-up, indicating survival, or had a recorded indication of death during the study follow-up, with no EMR data entries found afterward.

Another limitation of MELD-Plus is that it did not specifically consider which procedures patients underwent during the cirrhosis-related admissions. Furthermore, all the patients considered in our models survived the admission, but neither MGH/BWH’s nor IBM’s databases contained information on post-discharge cause of death. To further assess MELD-Plus’s applicability in clinical practice, future analyses should consider subgroups of patients to determine linkages between invasive inpatient procedures and causes of mortality. Regardless of this limitation, however, our MELD-Plus displayed validity in predicting overall mortality, which is clinically applicable, because it provides clinicians with information on populations of patients who need more intense or closer care.

Although we excluded elective admissions for liver biopsy, radiofrequency ablation, transarterial chemoembolization, hepatic resection, or liver transplant, these criteria might exclude patients with early and intermediate hepatocellular carcinoma (HCC), but not patients with advanced HCC who underwent medical treatments only. Liver cancer can lead to early mortality, even in patients with mild liver cirrhosis, and, as such, our exclusion criteria may reduce the applicability of our risk score when applying it to patients with more advanced HCC. Furthermore, because we excluded admissions associated with a liver transplant, mortality risk may decrease after a cirrhosis-related admission if patients successfully underwent a transplant in a preceding admission.

Another limitation of our study is algorithmic. The adaptive LASSO method identified 9 predictors and left out variables that may also be correlated with predicting death. Feature selection algorithms are known to be blind to the clinical importance of variables, and when highly correlated predictors are identified, the algorithm randomly selects one. On the one hand, important variables such as ascites, hepatocellular carcinoma, and diuretic medications were not selected as predictors. On the other hand, the feature selection algorithm assures that a minimal set of covariates produce a high level of prediction accuracy. Furthermore, we conducted our model performance evaluation on a held-out data set not used for training. Although a prediction model’s error usually decreases when more variables are included, this is not always the case. This is true when performance is evaluated on the training set (due to overfitting) but not the case when performance is evaluated on a held-out test dataset, as was used across all our models.

In conclusion, we describe an unbiased and well-validated score to estimate 90-day mortality after a cirrhosis-related admission. This score, comprising a small set of easily available clinical variables extracted from EMRs, improved the MELD and MELD-Na scores in predicting 90-day mortality and approximate 1-year mortality. In addition, we identified high-risk patients with great accuracy. MELD-Plus’s strong performance demonstrates potential for it to replace current standard models, allowing for greater accuracy in the identification of high-risk cirrhosis patients.

## Supporting information

S1 Table Summary of variables. (DOCX)

S2 Table Billing codes used to define conditions. (DOCX)

S3 Table Comparison of variables in patients who died vs. survived 90-days after discharge. (DOCX)

S4 Table Comparison between the highest-risk (1^*st*^) and the lowest-risk (5^*th*^) quintiles. (DOCX)

## Abbreviations

AUROC: Area under the receiver operating characteristic curve
CI: Confidence interval
COPD: Chronic obstructive pulmonary disease
CPT: Current procedural terminology
EMR: Electronic medical record
GGT: Gamma glutamyl transpeptidase
HE: Hepatic encephalopathy
HIPAA: Health Insurance Portability and Accountability Act
OR: Odds ratio
ICD-9-CM: International Classification of Diseases, Ninth Revision, Clinical Modification
LASSO: Least absolute shrinkage and selection operator
MELD: Model for end-stage liver disease
NAFLD: Nonalcoholic fatty liver disease
Na: Sodium’s chemical element symbo
NS: Not significant
SBP: Spontaneous bacterial peritonitis
SE: Standard error
STD: Standard deviation
WBC: White blood cell count
eGFR: Estimated glomerular filtration rate
