# Supplementary material for: The MELD-Plus: A generalizable prediction risk score in cirrhosis
Source: PLoS One. 2017 Oct 25;12(10):e0186301. doi: 10.1371/journal.pone.0186301 (PMC5656314; doi:10.1371/journal.pone.0186301)
Supplement: S1 Table — (DOCX) [file pone.0186301.s001.docx]

**S1 Table. Summary of variables.** All values extracted during the twelve months preceding discharge date. For laboratory variables, values are the most recent; for medication and comorbidity variables, values reflect the count of EMR entries. Prevalence calculations consider admissions with at least one measurement for laboratories and at least one billing code for comorbidities.

| **Variable and category** | **Cirrhosis-related admissions**  **(n = 4,781 admissions)** |
| --- | --- |
| **Age (years); Mean (SD)** | 60.0 (13.7) |
| **Gender (%)** | |
| Male | 64.4 |
| Female | 35.6 |
| **Ethnicity (%)** | |
| Caucasian | 77.4 |
| African American | 6.8 |
| Other | 2.3 |
| Unknown | 13.5 |
| **Marital Status (%)** |  |
| Married or partner | 35.7 |
| Other | 59.9 |
| Unknown | 4.3 |
| **Insurance Type (could be ≥ 1 types per patient) (%)** | |
| Medicaid | 5.6 |
| Medicare | 60.0 |
| Other | 98.6 |
| **MELD score; Mean (SD)** | 14.21 (6.1) |
| **NAFLD Fibrosis score; Mean (SD)** | 1.70 (2.1) |
| **BMI (kg/m^2^); Mean (SD)** | 28.7 (8.2) |
| **Length of Stay (days); Mean (SD)** | 7.3 (8.2) |
| **# Admissions (Any) Preceding 12 months** | 1.8 (2.3) |
| **Laboratory values; Mean (SD) / Prevalence (%)** | |
| Albumin (g/dl) | 2.9 (0.7) / 98.7 |
| Cholesterol (mg/dl) | 133.6 (50.7) / 50.8 |
| Creatinine (mg/dl) | 1.4 (1.2) / 99.8 |
| CRP (mg/l) | 43.9 (58.1) / 14.6 |
| eGFR (ml/min/1.73m^2^) | 59.6 (33.7) / 31.9 |
| ESR (mm/hr) | 52.3 (34.7) / 31.2 |
| Ferritin (ng/ml) | 418.5 (1880.0) / 58.1 |
| Fibrinogen (mg/dl) | 295.3 (160.1) / 20.2 |
| GGT (u/l) | 249.4 (346.6) / 6.8 |
| Globulin (g/dl) | 3.8 (1.0) / 98.0 |
| HDL Cholesterol (mg/dl) | 37.3 (19.4) / 46.8 |
| HGB A1C (%) | 6.8 (2.0) / 46.1 |
| LDL Cholesterol (mg/dl) | 74.8 (40.2) / 44.8 |
| Platelets (th/cumm) | 141.3 (100.0) / 99.8 |
| Prothrombin time (INR) | 1.5 (0.5) / 91.3 |
| Sodium (mmol/l) | 136.5 (5.8) / 99.7 |
| Total Bilirubin (mg/dl) | 2.5 (4.3) / 98.7 |
| Transaminase SGOT (u/l) | 60.2 (76.6) / 98.8 |
| Transaminase SGPT (u/l) | 36.1 (38.2) / 96.7 |
| Triglycerides (mg/dl) | 109.1 (75.4) / 49.7 |
| TSH (uu/ml) | 3.2 (5.9) / 54.1 |
| WBC (th/cumm) | 6.7 (3.8) / 99.8 |
| **Comorbidities; Mean (SD) / Prevalence (%)** |  |
| Variceal hemorrhage / Gastrointestinal bleed | 0.8 (2.1) / 24.2 |
| Spontaneous bacterial peritonitis | 0.1 (0.6) / 3.4 |
| Hepatocellular carcinoma | 0.7 (5.5) / 4.3 |
| Hepatorenal syndrome | 0.1 (0.5) / 3.2 |
| Hepatic encephalopathy | 1.4 (3.6) / 28.6 |
| Ascites | 2.4 (5.7) / 37.9 |
| Renal failure | 1.8 (8.4) / 13.1 |
| Cerebrovascular disease | 0.5 (2.4) / 10.8 |
| Congestive heart failure | 2.7 (7.9) / 36.7 |
| Hypertension | 3.1 (7.2) / 58.9 |
| Acute myocardial infarction | 0.5 (2.1) / 13.4 |
| Ischemic heart disease | 0.3 (1.2) / 12.4 |
| Peripheral vascular disease | 0.5 (2.2) / 13.0 |
| Diabetes | 5.9 (10.3) / 57.0 |
| Asthma | 0.5 (2.2) / 12.3 |
| Pneumonia | 1.4 (3.5) / 30.5 |
| COPD | 1.2 (4.6) / 21.1 |
| Atrial fibrillation / Atrial flutter | 1.1 (4.1) / 17.2 |
| Anxiety or depression | 0.5 (1.9) / 15.3 |
| Psychiatric disorder | 1.2 (4.5) / 21.5 |
| Sleep apnea | 0.1 (0.9) / 4.3 |
| Hepatic encephalopathy | 1.4 (3.6) / 28.6 |
| Asthma | 0.5 (2.2) / 12.3 |
| Disorders of lipid metabolism | 0.7 (2.1) / 22.6 |
| Obesity | 0.3 (1.4) / 10.9 |
| Joint disorder | 2.0 (5.0) / 35.9 |
| Gastrointestinal disorder | 1.2 (2.1) / 40.2 |
| Chronic kidney disease | 1.7 (9.6) / 12.8 |
| **Medications; Mean (SD)** |  |
| Anti-coagulants | 1.3 (2.7) |
| Anti-platelets | 0.2 (0.9) |
| Anti-arrhythmics and diuretics | 4.3 (6.2) |
| Aspirin | 1.9 (3.9) |
| Cardiovascular | 6.8 (8.9) |
| Diabetes | 2.4 (5.4) |
| Hepatic encephalopathy | 0.6 (1.7) |
| Hormones | 0.04 (0.4) |
| Lipid lowering | 0.8 (2.1) |
| Vitamin E | 0.04 (0.3) |
