# Supplementary material for: The MELD-Plus: A generalizable prediction risk score in cirrhosis
Source: PLoS One. 2017 Oct 25;12(10):e0186301. doi: 10.1371/journal.pone.0186301 (PMC5656314; doi:10.1371/journal.pone.0186301)
Supplement: S2 Table — (DOCX) [file pone.0186301.s002.docx]

**S2 Table. Billing codes used to define conditions**

| **Comorbidity** | **ICD9 Diagnosis Codes** | **ICD9 Procedure Codes** | **CPT Codes** |
| --- | --- | --- | --- |
| **Alcoholic cirrhosis** | 571.2, 571.3 | - | - |
| **Anxiety or depression** | 296.2, 296.3, 300.x | - | - |
| **Ascites** | 789.5 | - | - |
| **Asthma** | 493.x | - | - |
| **Atrial fibrillation /**  **Atrial flutter** | 427.31, 427.3, 427.32 | 37.33, 37.34 | 93653 to 93657, 33254 to 33259, 33265 to 33266 |
| **Biliary cirrhosis** | 571.6 | - | - |
| **Cerebrovascular disease** | 430.x, 431.x, 432.x, 433.x, 436 | - | - |
| **Chronic kidney disease** | 585.1, 585.2, 585.3, 585.4, 585.5, 585.6, 585.9 | - | - |
| **Cirrhosis** | 571.2, 571.5, 571.6 | - | - |
| **Cirrhosis of liver without alcohol** | 571.5 | - | - |
| **Cirrhosis secondary to hepatitis B or C** | 070.44, 070.71, 070.6, 070.33, 070.0, 070.1, 070.54, 070.70, 070.49, 070.59, 070.9, 571.49, 571.41 | - | - |
| **Congestive heart failure** | 428.x | - | - |
| **COPD** | 491.x, 492.x, 496 | - | - |
| **Ischemic heart disease** | 410, 411, 412, 413, 414 | 36, 00.66 | 92995, 92996, 92982, 92984, 92980, 92981, 33510 to 33545 |
| **Acute myocardial infarction** | 410.x | - | - |
| **Diabetes** | 250.x | - | - |
| **Disorders of lipid metabolism** | 272, 272.0, 272.1, 272.2, 272.3, 272.4, 272.5, 272.6, 272.7, 272.8, 272.9 | - | - |
| **Gastrointestinal disorder** | Duodenal Ulcer: 532.x  Esophageal Reflux: 53081  Esophagitis: 5301  Gastric Ulcer: 531.x  Gastritis and Duodenitis: 535.x  Gastrojejunal Ulcer: 534.x  Peptic Ulcer: 533.x  Ulcer of Esophagus: 5302 | - | - |
| **Hepatic encephalopathy** | 572.2 | - | - |
| **Hepatic resection** | 50.22 | - | - |
| **Hepatocellular carcinoma** | 155.0 | - | - |
| **Hepatorenal syndrome** | 572.4 | - | - |
| **Hypertension** | 401.x, 997.91 | - | - |
| **Joint disorder** | Crystal Arthropathies: 712.x  Rheumatoid Arthritis and Other Inflammatory Polyarthropathies: 714.x  Dorsopathies: 720.x, 721.x, 722.x, 723.x, 724.x  Internal Derangement of Knee: 717.x  Osteoarthrosis and Allied Disorder: 715.x  Psoriatic Arthropathy: 696.0 | - | - |
| **Liver biopsy** | 50.11, 50.12, 50.14, 50.19 | - | - |
| **Liver transplant** | 99682, V42.7 | 50.51, 50.5, 50.59 | - |
| **NAFLD** | 571.8, 571.9 | - | - |
| **Obesity** | 278, 278.0, 278.00, 278.01, 278.02 | - | - |
| **Peripheral vascular disease** | 443.9, 785.4, 250.7 443.81, 440.21, 440.22, 440.23 | 84.1x, 00.55, 39.90, 00.60, 39.22, 39.24, 39.25, 39.26, 39.50, 38.13, 38.18, 00.40 to 00.43, 00.46 to 00.48 | 37220, 37222, 37224, 37228, 37232, 37205, 37221, 37223, 37226, 37230, 37234, 27590 to 27598, 28800, 28805, 28810, 28820, 28825, 27880, 27881, 27882, 27884, 27886, 28888, 27889, 27290, 27295 |
| **Pneumonia** | 480.x, 481.x, 482.x, 483.x, 484.x, 485.x, 486.x, 487.0, 488.01, 488.11 | - | - |
| **Psychiatric disorder** | 294.0, 294.8, 294.9, 295.x, 296.x, 297.x 298.x (excluding 296.2, 296.3) | - | - |
| **Radiofrequency ablation** | 50.2, 50.23, 50.24, 50.25, 50.29 | - | - |
| **Renal failure** | 250.4, 585, 583.81, 581.81 | 00.91, 00.92, 00.93, 39.95, 54.98 | 90935, 90937, 90945, 90947, 90999, 50360, 50365 |
| **Sleep apnea** | 327.23, 327.2, 327.29, 780.57, 327.20, 327.21, 780.51, 780.53 | - | - |
| **Spontaneous bacterial peritonitis** | 567.23 | - | - |
| **Transarterial chemoembolization** | 50.94 | - | - |
| **Variceal hemorrhage /**  **Gastrointestinal bleed** | 456.0, 456.1 | - | - |
