# Supplementary material for: The MELD-Plus: A generalizable prediction risk score in cirrhosis
Source: PLoS One. 2017 Oct 25;12(10):e0186301. doi: 10.1371/journal.pone.0186301 (PMC5656314; doi:10.1371/journal.pone.0186301)
Supplement: S3 Table — (DOCX) [file pone.0186301.s003.docx]

**S3 Table. Comparison of variables in patients who died vs. survived 90-days after discharge.** All values extracted during the twelve months preceding discharge date. For laboratory variables, values reflect the average of the most recent value. For comorbid and medication variables, values reflect the average count of EMR entries. Using a Bonferroni correction, the adjusted *P* value was 7.4∙10^-4^ for each comparison.

| **Variable** | **Survived**  **(n = 4,003 admissions)** | **Deceased**  **(n = 778 admissions)** | ***P* value** |
| --- | --- | --- | --- |
| **MELD Score** | 13.3 | 18.5 | 4.45∙10^-65^‡ |
| **Total Bilirubin** | 2.02 | 4.87 | 1.82∙10^-41^‡ |
| **Creatinine** | 1.29 | 1.80 | 1.24∙10^-36^‡ |
| **Albumin** | 2.95 | 2.60 | 5.64∙10^-35^‡ |
| **Prothrombin time (INR)** | 1.46 | 1.70 | 5.63∙10^-30^‡ |
| **Ascites** | 2.0 | 4.4 | 3.55∙10^-24^‡ |
| **WBC** | 6.4 | 8.2 | 2.25∙10^-23^‡ |
| **Hepatorenal Syndrome** | 0.04 | 0.19 | 4.60∙10^-21^‡ |
| **Length of Stay** | 6.9 | 9.5 | 2.17∙10^-20^‡ |
| **Age** | 59.2 | 64.1 | 4.22∙10^-17^* |
| **Hepatocellular Carcinoma** | 0.40 | 2.00 | 1.40∙10^-16^‡ |
| **Hepatic Encephalopathy** | 1.2 | 2.0 | 2.49∙10^-14^‡ |
| **Ferritin** | 403.7 | 486.5 | 6.85∙10^-14^‡ |
| **Chronic Kidney Disease / End Stage Renal Disease** | 1.5 | 2.5 | 7.07∙10^-14^‡ |
| **Anti-arrhythmics and Diuretics Meds** | 4.1 | 5.8 | 7.46∙10^-13^‡ |
| **Spontaneous Bacterial Peritonitis** | 0.07 | 0.21 | 2.16∙10^-11^‡ |
| **Sodium** | 136.8 | 135.3 | 2.95∙10^-10^‡ |
| **Cholesterol** | 136.0 | 121.3 | 7.92∙10^-10^‡ |
| **Renal Failure** | 1.6 | 2.7 | 6.86∙10^-9^‡ |
| **Hepatic Encephalopathy Meds** | 0.51 | 0.86 | 4.98∙10^-8^‡ |
| **HDL Cholesterol** | 38.4 | 31.6 | 5.74∙10^-8^‡ |
| **NAFLD Fibrosis Score** | 1.5 | 2.7 | 1.13∙10^-5^‡ |
| **eGFR** | 60.6 | 55.0 | 1.59∙10^-5^‡ |
| **Transaminase SGOT** | 58.1 | 71.2 | 2.87∙10^-5^‡ |
| **HGB A1C** | 6.9 | 6.3 | 7.70∙10^-5^‡ |
| **Marital Status: Other** | 0.61 | 0.53 | 0.000576581† |
| **Insurance: Other** | 0.99 | 0.97 | NS (0.00094894)† |
| **Platelets** | 144.2 | 126.4 | NS (0.002630533)‡ |
| **TSH** | 3.1 | 3.4 | NS (0.002757636)‡ |
| **Atrial fibrillation / Atrial flutter** | 1.1 | 1.4 | NS (0.003946903)‡ |
| **Congestive Heart Failure** | 2.5 | 3.6 | NS (0.009335963)‡ |
| **Disorders of Lipid Metabolism** | 0.77 | 0.59 | NS (0.010405546)‡ |
| **Gamma Glutamyl Transpeptidase** | 242.8 | 274.9 | NS (0.027069564)‡ |
| **# Admissions Prec. 12 Months** | 1.8 | 2.1 | NS (0.030302102)‡ |
| **Marital Status: Married or Partner** | 0.35 | 0.41 | NS (0.033009762)† |
| **Obesity** | 0.33 | 0.21 | NS (0.047915231)‡ |
| **Sleep Apnea** | 0.12 | 0.11 | NS (0.078274362)‡ |
| **Diabetes Meds** | 2.3 | 2.6 | NS (0.169081147)‡ |
| **Globulin** | 3.8 | 3.7 | NS (0.194519558)‡ |
| **Anxiety or Depression** | 0.50 | 0.29 | NS (0.302483506)‡ |
| **Vitamin E Meds** | 0.03 | 0.07 | NS (0.375861046)‡ |
| **Ethnicity: Other** | 0.02 | 0.04 | NS (0.409054821)† |
| **Asthma** | 0.50 | 0.28 | NS (0.475737057)‡ |
| **Triglycerides** | 110.8 | 100.5 | NS (0.515820389)‡ |
| **LDL Cholesterol** | 75.3 | 72.3 | NS (0.942468781)‡ |
| **Gender: Male** | 0.64 | 0.65 | NS (1.0)† |
| **Ethnicity: White** | 0.77 | 0.77 | NS (1.0)† |
| **Ethnicity: African American** | 0.07 | 0.07 | NS (1.0)† |
| **Insurance: Medicare** | 0.59 | 0.63 | NS (1.0)† |
| **Insurance: Medicaid** | 0.06 | 0.05 | 1 NS (1.0)† |
| **Variceal Hemorrhage or GI Bleed** | 0.82 | 0.68 | NS (1.0)‡ |
| **COPD** | 1.25 | 1.04 | NS (1.0)‡ |
| **Cerebrovascular disease** | 0.47 | 0.56 | NS (1.0)‡ |
| **Diabetes** | 6.0 | 5.7 | NS (1.0)‡ |
| **Gastrointestinal Disorder** | 1.19 | 1.15 | NS (1.0)‡ |
| **Hypertension** | 3.19 | 2.67 | NS (1.0)‡ |
| **Ischemic Heart Disease** | 0.34 | 0.32 | NS (1.0)‡ |
| **Joint Disorder** | 2.03 | 1.75 | NS (1.0)‡ |
| **Myocardial Infarction** | 0.47 | 0.57 | NS (1.0)‡ |
| **Peripheral Vascular Disease** | 0.48 | 0.52 | NS (1.0)‡ |
| **Pneumonia** | 1.35 | 1.44 | NS (1.0)‡ |
| **Psychiatric Disorder** | 1.30 | 0.83 | NS (1.0)‡ |
| **CRP** | 43.2 | 47.5 | NS (1.0)‡ |
| **ESR** | 52.3 | 52.2 | NS (1.0)‡ |
| **Fibrinogen** | 299.4 | 280.4 | NS (1.0)‡ |
| **Transaminase SGPT** | 36.0 | 36.5 | NS (1.0)‡ |
| **Anti-coagulants Meds** | 1.31 | 1.30 | NS (1.0)‡ |
| **Anti-platelets Meds** | 0.18 | 0.09 | NS (1.0)‡ |
| **Aspirin Meds** | 1.88 | 1.76 | NS (1.0)‡ |
| **Cardiovascular Meds** | 6.5 | 6.7 | NS (1.0)‡ |
| **Hormones Meds** | 0.04 | 0.04 | NS (1.0)‡ |
| **Lipid Lowering Meds** | 0.78 | 0.64 | NS (1.0)‡ |
| **BMI** | 28.7 | 28.7 | NS (1.0)* |
| † Chi-square test  * Student’s t-test  ‡ Wilcoxon rank sum test | | | |
