# Supplementary material for: The MELD-Plus: A generalizable prediction risk score in cirrhosis
Source: PLoS One. 2017 Oct 25;12(10):e0186301. doi: 10.1371/journal.pone.0186301 (PMC5656314; doi:10.1371/journal.pone.0186301)
Supplement: S4 Table — (DOCX) [file pone.0186301.s004.docx]

**S4 Table. Comparison between the highest-risk (1*^st^*) and the lowest-risk (5*^th^*) quintiles.** All values extracted during the twelve months preceding discharge date. For laboratory variables, values reflect the average of the most recent value preceding discharge date. For comorbid variables, values reflect the average count of EMR entries. Using a Bonferroni correction, the adjusted *P* value was 7.4∙10^-4^ for each comparison.

| **Variable** | **1*^st^* quantile**  **(n = 956 admissions)** | **5*^th^* quantile**  **(n = 957 admissions)** | ***P* value** |
| --- | --- | --- | --- |
| **Albumin** | 2.15 | 3.58 | 2.35∙10^-289^‡ |
| **WBC** | 10.14 | 3.92 | 2.64∙10^-239^‡ |
| **Sodium** | 134.01 | 137.94 | 5.91∙10^-50^‡ |
| **Length of Stay** | 9.57 | 5.32 | 6.76∙10^-48^‡ |
| **Total Bilirubin** | 3.92 | 1.53 | 1.18∙10^-47^‡ |
| **Ascites** | 3.48 | 1.24 | 1.41∙10^-42^‡ |
| **Platelets** | 167.25 | 110.59 | 6.32∙10^-39^‡ |
| **MELD Score** | 16.08 | 12.69 | 1.41∙10^-34^‡ |
| **Ferritin** | 718.57 | 260.64 | 8.79∙10^-29^‡ |
| **Prothrombin time (INR)** | 1.66 | 1.43 | 6.44∙10^-26^‡ |
| **Globulin** | 4.07 | 3.54 | 1.08∙10^-25^‡ |
| **Transaminase SGOT** | 73.54 | 51.18 | 1.48∙10^-22^‡ |
| **Disorders of lipid metabolism** | 0.43 | 1.16 | 1.00∙10^-21^‡ |
| **Hepatic encephalopathy** | 1.73 | 0.95 | 7.32∙10^-17^‡ |
| **Lipid Lowering Meds** | 0.35 | 1.11 | 8.22∙10^-16^‡ |
| **Ischemic Heart Disease** | 0.15 | 0.55 | 5.72∙10^-11^‡ |
| **ESR** | 61.29 | 42.99 | 5.40∙10^-8^‡ |
| **Anti-platelets Meds** | 0.03 | 0.33 | 6.29∙10^-8^‡ |
| **Aspirin Meds** | 1.24 | 2.41 | 9.67∙10^-8^‡ |
| **HDL Cholesterol** | 32.69 | 39.11 | 3.48∙10^-7^‡ |
| **Hypertension** | 2.88 | 3.67 | 5.78∙10^-7^‡ |
| **Diabetes Meds** | 1.90 | 2.93 | 1.06∙10^-5^‡ |
| **Anti-coagulants Meds** | 1.19 | 1.74 | 3.00∙10^-5^‡ |
| **Anti-arrhythmics and Diuretics Meds** | 4.32 | 4.12 | 9.48∙10^-5^‡ |
| **Anxiety and Depression** | 0.23 | 0.71 | 0.000156‡ |
| **CRP** | 61.39 | 33.54 | 0.000166‡ |
| **Sleep apnea** | 0.08 | 0.14 | NS (0.001697)‡ |
| **Cardiovascular Meds** | 5.54 | 7.13 | NS (0.003501)‡ |
| **Ethnicity: African American** | 0.11 | 0.06 | NS (0.004213)† |
| **Pneumonia** | 1.61 | 1.13 | NS (0.004766)‡ |
| **Hepatorenal syndrome** | 0.09 | 0.03 | NS (0.023386)‡ |
| **Gender: Male** | 0.70 | 0.62 | NS (0.031378)† |
| **Gamma Glutamyl Transpeptidase** | 358.44 | 161.14 | NS (0.053248)‡ |
| **Diabetes** | 5.28 | 6.61 | NS (0.086615)‡ |
| **Chronic Kidney Disease / End Stage Renal Disease** | 1.37 | 1.50 | NS (0.18795)‡ |
| **Triglycerides** | 105.64 | 116.27 | NS (0.263208)‡ |
| **Psychiatric Disorder** | 0.83 | 1.72 | NS (0.328907)‡ |
| **Hormones Meds** | 0.01 | 0.05 | NS (0.368661)‡ |
| **Renal Failure** | 2.18 | 1.23 | NS (0.432317)‡ |
| **Obesity** | 0.25 | 0.38 | NS (0.581007)‡ |
| **Spontaneous bacterial peritonitis** | 0.10 | 0.06 | NS (0.587747)‡ |
| **Transaminase SGPT** | 37.95 | 35.97 | NS (0.715784)‡ |
| **Ethnicity: White** | 0.74 | 0.79 | NS (0.765123)† |
| **Age** | 59.23 | 59.16 | NS (1.0)* |
| **Marital Status: Other** | 0.59 | 0.60 | NS (1.0)† |
| **Marital Status: Married or Partner** | 0.36 | 0.37 | NS (1.0)† |
| **Insurance: Medicare** | 0.58 | 0.63 | NS (1.0)† |
| **Insurance: Medicaid** | 0.07 | 0.05 | NS (1.0)† |
| **Insurance: Other** | 0.99 | 0.98 | NS (1.0)† |
| **Variceal Hemorrhage or GI Bleed** | 0.69 | 0.82 | NS (1.0)‡ |
| **Hepatocellular carcinoma** | 1.46 | 0.44 | NS (1.0)‡ |
| **Asthma** | 0.47 | 0.35 | NS (1.0)‡ |
| **Atrial fibrillation / Atrial flutter** | 0.83 | 1.17 | NS (1.0)‡ |
| **CHF** | 1.80 | 2.91 | NS (1.0)‡ |
| **COPD** | 1.15 | 0.89 | NS (1.0)‡ |
| **CVD** | 0.40 | 0.50 | NS (1.0)‡ |
| **Gastronomical Disorder** | 1.19 | 1.25 | NS (1.0)‡ |
| **Joint Disorder** | 1.66 | 2.55 | NS (1.0)‡ |
| **Myocardial Infarction** | 0.27 | 0.66 | NS (1.0)‡ |
| **Peripheral vascular disease** | 0.41 | 0.54 | NS (1.0)‡ |
| **Cholesterol** | 133.11 | 133.49 | NS (1.0)‡ |
| **eGFR** | 66.04 | 57.62 | NS (1.0)‡ |
| **Fibrinogen** | 286.17 | 308.69 | NS (1.0)‡ |
| **LDL Cholesterol** | 79.23 | 70.58 | NS (1.0)‡ |
| **TSH** | 3.09 | 3.08 | NS (1.0)‡ |
| **Creatinine** | 1.36 | 1.33 | NS (1.0)‡ |
| **HGB A1C** | 6.75 | 6.77 | NS (1.0)‡ |
| **Hepatic Encephalopathy Meds** | 0.53 | 0.61 | NS (1.0)‡ |
| **Vitamin E Meds** | 0.05 | 0.02 | NS (1.0)‡ |
| **BMI** | 28.31 | 28.72 | NS (1.0)* |
| **NAFLD Fibrosis Score** | 3.41 | 3.58 | NS (1.0)‡ |
| **# Admissions Prec. 12 Months** | 1.80 | 1.89 | NS (1.0)‡ |
| † Chi-square test  * Student’s t-test  ‡ Wilcoxon rank sum test | | | |
